# Neutrophil to High-density Lipoprotein ratio (NHR) as a potential predictor of disease severity and survival time in Creutzfeldt-Jakob disease

**DOI:** 10.1186/s12883-023-03076-y

**Published:** 2023-01-23

**Authors:** Yu Kong, Zhongyun Chen, Jing Zhang, Liyong Wu

**Affiliations:** Department of Neurology, Xuanwu Hospital, Capital Medical University, Beijing, China

**Keywords:** Creutzfeldt-Jakob disease, Neutrophil to lymphocyte ratio, High-density lipoprotein, Monocyte to high-density lipoprotein, Neutrophil to high-density lipoprotein, Severity, Survival time

## Abstract

**Introduction:**

Creutzfeldt-Jakob disease (CJD) is a fatal and irreversible neurodegenerative disease. Identification of inexpensive and easy-to-implement biomarkers of CJD which could predict disease severity and patient survival is important for improving disease management. The aim of this study was to assess the predictive value of peripheral neutrophil to lymphocyte ratio (NLR), high-density lipoprotein (HDL), monocyte to HDL ratio (MHR) and neutrophil to HDL ratio (NHR) for CJD.

**Methods:**

Patients with definite or probable CJD admitted to the Neurology Department of Xuanwu Hospital from 2014 to 2021 were enrolled and followed up until April 2022. Clinical information including sex, age, Barth Index, survival time and results of auxiliary examination were collected, and NLR, HDL, NHR and MHR were measured for all enrolled patients. The associations between NLR, HDL, NHR and MHR, and disease severity (evaluated by Barth Index), survival time and auxiliary examinations were evaluated.

**Results:**

A total of 88 CJD patients were enrolled and all were deceased. NLR (*r* = -0.341, *p* = 0.001), NHR (*r* = -0.346, *p* = 0.001) and MHR (*r* = -0.327, *p* = 0.002) were significantly associated with disease severity. Higher NHR (HR = 2.344, 95% CI = 1.277–4.303 *p* = 0.006) and lower HDL (HR = 0.567, 95% CI = 0.346–0.930, *p* = 0.025) were associated with shorter survival time in the CJD patients.

**Conclusions:**

Peripheral inflammatory biomarkers, especially NHR, were associated with disease severity and survival duration. These findings provide new insights into the mechanisms and treatment strategies of CJD.

**Supplementary Information:**

The online version contains supplementary material available at 10.1186/s12883-023-03076-y.

## Introduction

Creutzfeldt-Jakob disease (CJD) is a fatal and irreversible neurodegenerative disease caused by the accumulation of abnormal prion protein in the central nervous system (CNS). The survival time ranges from weeks to several years, with an average of 5 to 6 months [1, 2]. There are currently no drugs or other interventions that can reverse or prevent the progression of symptoms and neurodegeneration associated with CJD. Therefore, the discovery of prognosis-related indicators could considerably benefit disease management and health care through the prognostic stratification of patients. The well-established prognostic biomarkers of CJD, such as 14–3-3 protein, phosphorylated-tau (P-tau) and total tau (T-tau) in the cerebrospinal fluid (CSF), are relatively expensive and difficult to detect and therefore cannot be implemented in a large-scale manner [2]. Thus, it is critical to identify cost-effective and convenient biomarkers of CJD. Peripheral blood biomarkers in particular are highly suitable for clinical applications.

Studies increasingly show that neuroinflammation is the cardinal pathological basis of CJD [3, 4]. Furthermore, increased levels of inflammatory proteins such as C-reactive protein (CRP), interleukin (IL-6), and α1-antitrypsin (AAT) in the plasma of CJD patients also suggest the involvement of peripheral inflammatory responses [5–7]. However, it remains unclear whether peripheral inflammatory biomarkers are associated with disease severity and progression of CJD. The neutrophil-to-lymphocyte ratio (NLR), an combined indicator of peripheral inflammation, is a robust prognostic biomarker of neurodegenerative diseases such as Alzheimer’s disease (AD), Parkinson’s disease (PD), amyotrophic lateral sclerosis (ALS) and multiple system atrophy (MSA), as well as a measure of disease severity [8–10]. High-density lipoprotein (HDL) is the predominant lipoprotein in the human brain, and exhibits antioxidant and anti-inflammatory effects [11, 12]. Studies show that HDL protects against pathological changes in the brain and cognitive decline, and lower levels of HDL were associated with a higher risk of neuronal degeneration [13, 14]. Furthermore, monocyte to high-density lipoprotein ratio (MHR) and neutrophil to high-density lipoprotein ratio(NHR), which are indicative of the anti-inflammatory and antioxidant effects of HDL and the pro-inflammatory effects of the immune cells, also have prognostic value in neurological diseases [15–18]. These peripheral inflammatory biomarkers can be easily incorporated in routine clinical tests given the simple detection methods. However, their ability to predict disease severity and prognosis of CJD has never been studied.

Thus, the aim of this study is to investigate the correlation between NLR, HDL, MHR, NHR, disease severity and survival in CJD patients.

## Methods

### Ethics statement

The ethics committee of Xuanwu Hospital, Capital Medical University approved the study. All participants and/or their legal guardians signed the written informed consent. All methods were carried out in accordance with relevant guidelines and regulations.

### Participants

**Fig. 1:**
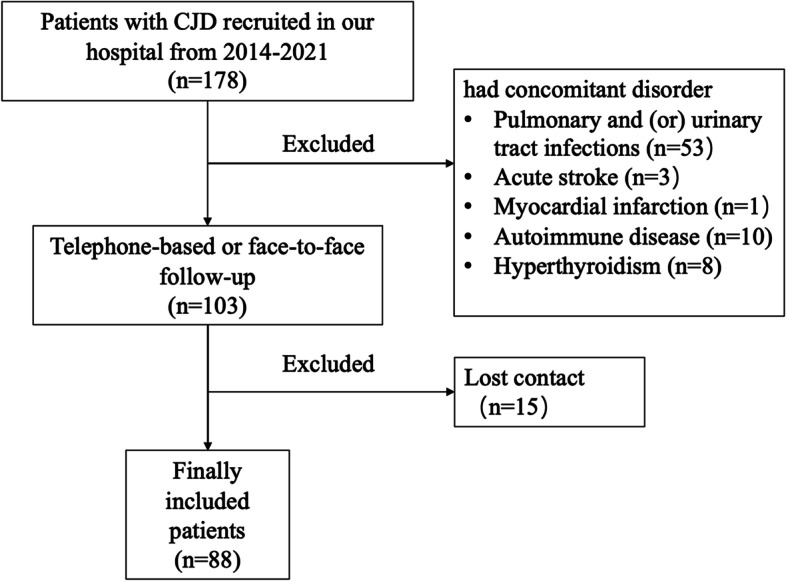
Flow diagram of patient inclusion

All participants included in this study referred to the Department of Neurology, Xuanwu Hospital, Capital Medical University between January 2014 to April 2021, and were diagnosed with definite or probable CJD according to the established guidelines [19, 20]. Brain magnetic resonance imaging (MRI) scans were performed to exclude other neurological disorders. Patients with pulmonary infections, urinary tract infections, acute stroke, myocardial infarction, malignant neoplasms, autoimmune disease, chronic inflammatory, hyperthyroidism, severe liver and kidney damage and those undergoing steroids therapy were excluded. Patients were also excluded if blood test results were not available. Telephone-based or face-to-face follow-ups were conducted until patients were deceased or until April 2022. Patients lost during follow-up were excluded. Finally, 88 patients with CJD were included in the final analysis. The patient selection process is outlined in Fig. 1.

### Clinical data collection

Clinical information including sex, age, disease severity, survival time and results of auxiliary examination (neuron-specific enolase [NSE] levels in plasma, 14–3-3 protein in CSF, electroencephalography [EEG] and MRI) were collected for all enrolled patients. The disease severity was quantified by Barth Index [21, 22]. Survival time was defined as the time between the date of initial symptom onset to the date of death. MRI scans were conducted on a 3.0 Tesla MRI system (Siemens Magnetom Trio Tim MRI system, Germany) using standard coil. The high signal of DWI or FLAIR in caudate/putamen or at least two cortical regions (temporal, parietal or occipital) were indicative of CJD. Since previous studies have shown the presence of diffusion restriction in basal ganglia in the later stage of CJD and correlated it with faster disease progression [23, 24], we also considered basal ganglia hyperintensity as a marker of more severe disease stage. EEG was performed using a 32-channel digital EEG system (DAVINCI-SAM, Micromed, Mogliano Veneto, Italy). Periodic sharp wave complexes (PSWCs) were described as the typical EEG pattern. CSF 14–3-3 protein was detected by western blotting as described previously [25]. *PRNP* gene and polymorphism of codon 129 were also tested [26].

### Blood tests and biochemical analysis

Blood was drawn from the cubital vein of the participants in the morning after overnight fasting for at least 8 h, and sent to our hospital lab for testing within one hour. Neutrophil, lymphocyte and monocyte counts were determined by standard laboratory methods, and HDL was measured using a Hitachi 7600 automatic biochemical analyzer (Hitachi, Tokyo, Japan). The normal range of neutrophils, lymphocytes, monocytes and HDL concentration are 1.8–6.4 × 10^9^/L, 1–3.3 × 10^9^/L, 0.2–0.7 × 10^9^/L and 1.08–1.91 mmol/L respectively. The NLR was obtained by dividing the neutrophil count by the monocyte count, NHR by dividing the neutrophil count by HDL levels, and MHR by the dividing monocytes count by HDL levels. The detection of inflammatory biomarkers and assessment of Barth index were performed on the same day.

### Statistical analyses

All analyses were conducted using SPSS 25.0. Categorical variables are shown as the frequency (percentage). Continuous variables are shown as the mean (± standard deviation[SD]) or median and interquartile range (IQR) depending on whether they conformed to normal distribution. T-test or Mann–Whitney U test was used for intergroup comparisons when appropriate. The associations between inflammatory biomarkers and disease severity were assessed with Pearson’s or Spearman’s rank correlation analyses depending on whether the parameters were normally distributed. Univariate and multivariate survival analyses were performed using Cox proportional hazard models. A two-sided *p* < 0.05 was considered statistically significant.

## Results

### Characteristics of participants

A total of 88 participants were enrolled in the final analysis, and almost one-third of the patients were female. The median (IQR) age was 62 (57–66) years. The majority cases (*n* = 86) were sporadic CJD, while only two cases were genetic CJD. The polymorphism of codon 129 was methionine homozygote (MM) in all 88 patients. All patients were deceased at the time of data analysis. The clinical and inflammatory biomarkers data are summarized in Table 1.

**Table 1:** Clinical and inflammatory biomarkers data of participants

Characteristic	Overall (*n* = 88)
Age (y), median (IQR)	62.0 (57.0–66.0)
Sex (Male/Female)	59/29
Barth Index, median (IQR)	45.0 (20.0–75.0)
Survival time (m), median (IQR)	12.5 (5.0–25.8)
NSE in plasma (ng/ml), median (IQR)	18.4 (14.3–26.1)
PSWCs on EEG, positive/total *n* (%)	22/81 (27.2%)
Hyperintense of basal ganglia on MRI, positive/total *n* (%)	22/88 (25%)
CSF 14–3-3 protein, positive/total *n* (%)	36/75 (48%)
Inflammatory	
NLR, median (IQR)	2.0 (1.5–2.6)
HDL (mmol/L), mean (± SD)	1.4 (± 0.4)
NHR, median (IQR)	2.5 (1.7–3.3)
MHR, median (IQR)	0.2 (0.2–0.3)

Continuous data were presented as the mean (± standard deviation[SD]) for normally distributed data and as median (interquartile range[IQR]) for non-normally distributed data. Categorical data were described as the frequency/total n (percentage)

*NSE* Neuron-specific enolase, *PSWCs* Periodic sharp wave complexes, *NLR* Neutrophil-to-lymphocyte ratio, *HDL* High-density lipoprotein, *NHR* Neutrophil to high-density lipoprotein ratio, *MHR* Monocyte to high-density lipoprotein ratio

### Association between inflammatory biomarkers and clinical characteristics

**Fig. 2:**
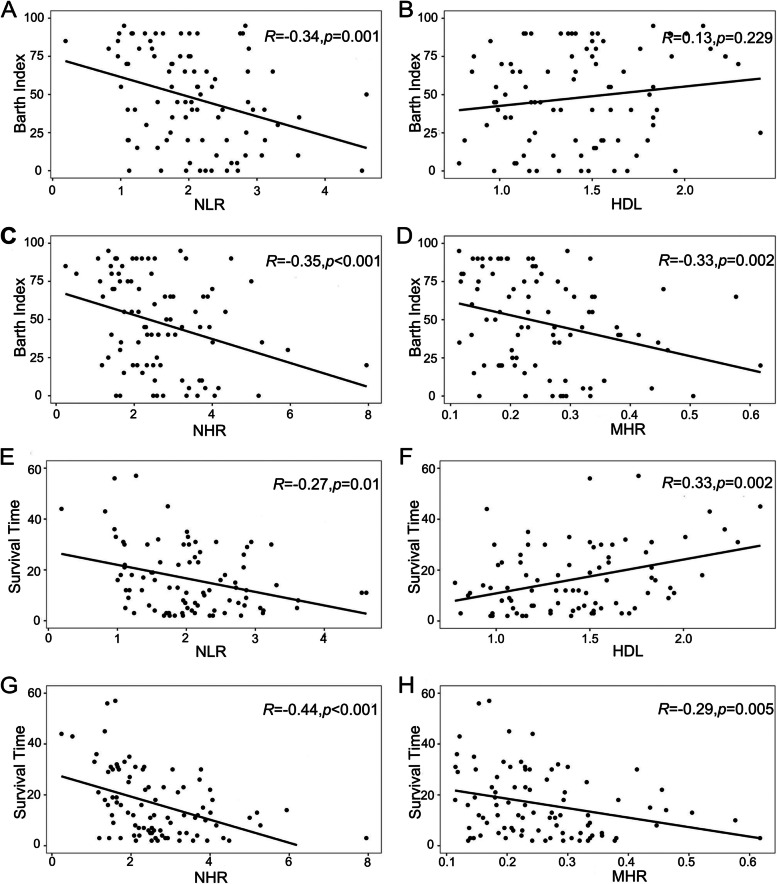
The association between inflammatory biomarkers and disease severity and survival. **a-d** Correlation between NLR (a), HDL (b), NHR (c) and MHR (d) and Barth Index. **e–h** Correlation between NLR (e), HDL (f), NHR (g) and MHR (h) and survival time. NLR, neutrophil-to-lymphocyte ratio; HDL, high-density lipoprotein; NHR, neutrophil to high-density lipoprotein ratio; MHR, monocyte to high-density lipoprotein ratio

HDL was higher in female patients compared to males (1.56[± 0.38] mmol/L vs 1.35[± 0.34] mmol/L, *p* = 0.011), while the NHR was higher in males than in females (2.52 [1.97–3.57] vs 1.83[1.42–3.28], *p* = 0.034). No significant correlation was observed between age and these biomarkers. The NLR (*r* = -0.341, *p* = 0.001), NHR (*r* = -0.346, *p* = 0.001) and MHR (*r* = -0.327, *p* = 0.002) showed a significant negative correlation with the Barth Index. Furthermore, the survival time was negatively correlated to NLR (*r* = -0.273, *p* = 0.01), NHR (*r* = -0.443, *p* < 0.001) and MHR (*r* = -0.294, *p* = 0.005) and positively with HDL (*r* = 0.331, *p* = 0.002) (Table 2 and Fig. 2).

**Table 2:** The association between inflammatory biomarkers and disease severity and prognosis

Inflammatory biomarkers	Correlation with Barth Index, *r*	*p* value	Correlation with survival time, *r*	*p* value
NLR	-0.341	0.001	-0.273	0.01
HDL	0.129	0.229	0.331	0.002
NHR	-0.346	0.001	-0.443	< 0.001
MHR	-0.327	0.002	-0.294	0.005

*NLR* Neutrophil-to-lymphocyte ratio, *HDL* High-density lipoprotein, *NHR* Neutrophil to high-density lipoprotein ratio, *MHR* Monocyte to high-density lipoprotein ratio

**Fig. 3:**
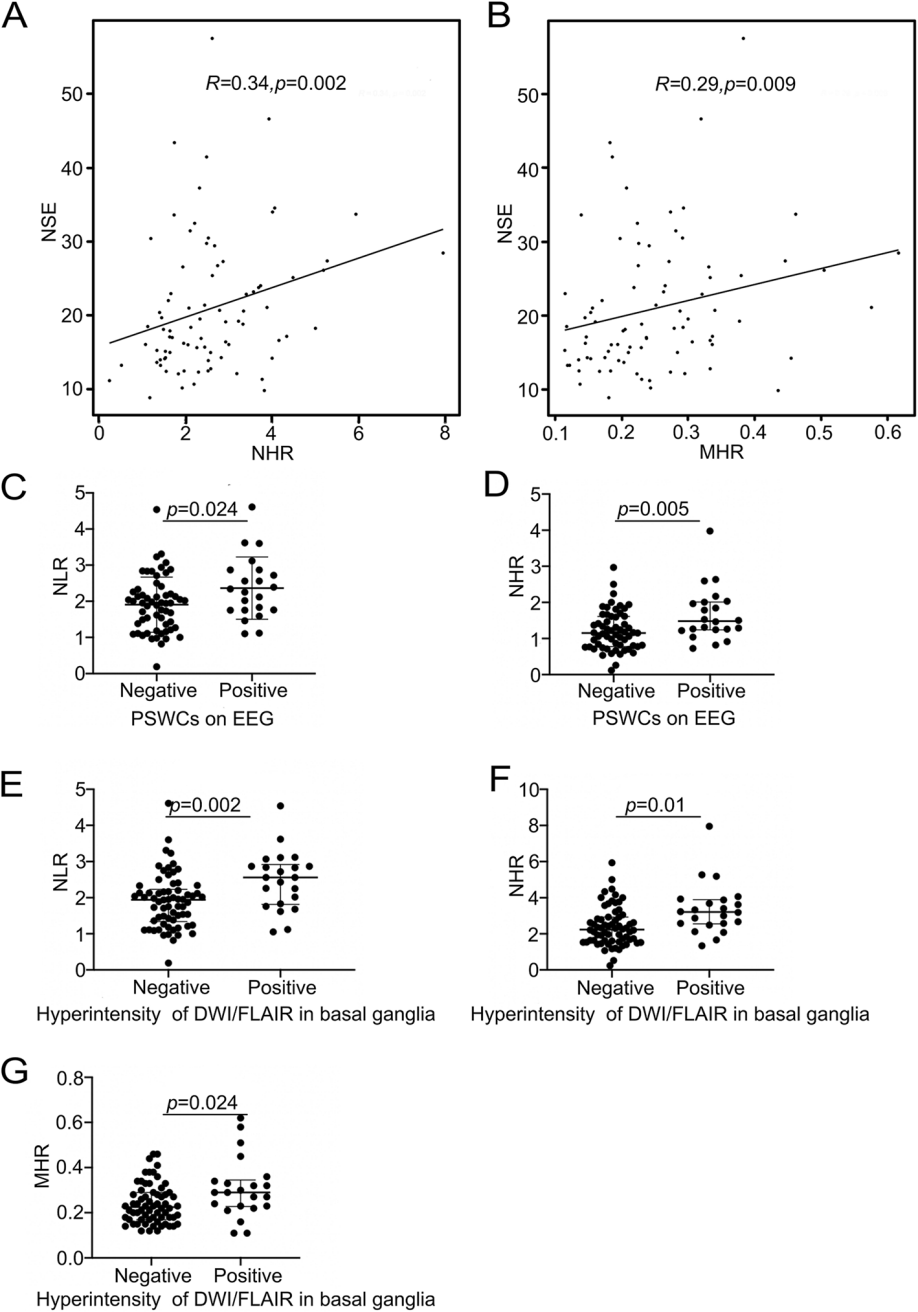
Correlation between inflammatory biomarkers and auxiliary examinations. **a**, **b** The relationship between NHR and MHR and NSE. **c**, **d** The comparison of NLR and NHR between patients with PSWCs on EEG and patients without PSWCs. (e–g) The comparison of NLR, NHR and MHR between patients with hyperintensity of DWI/FLAIR in basal ganglia and patients without hyperintensity of DWI/FLAIR in basal ganglia. NSE, neuron-specific enolase; PSWCs, periodic sharp wave complexes; NLR, neutrophil-to-lymphocyte ratio; HDL, high-density lipoprotein; NHR, neutrophil to high-density lipoprotein ratio; MHR, monocyte to high-density lipoprotein ratio

In addition, NLR, NHR and MHR were significantly higher in patients with hyperintensity in the basal ganglia on MRI compared to those without the involvement of basal ganglia. NLR and NHR in patients with PSWCs were also significantly higher compared to those without PSWCs. In addition, NHR and MHR were positively correlated to peripheral NSE. The data are presented in Fig. 3 and Supplemental Table 1.

### Association of inflammatory biomarkers and survival of CJD patients

All patients died at the end of the follow-up period, and the prognostic factors of survival were explored by univariate and multivariate Cox proportional hazards regression analyses. NLR, HDL, NHR and MHR were demarcated into “high” and “low” based on their median values. The univariate model showed that higher levels of NHR (HR = 3.206, 95% CI = 1.967–5.223, *p* < 0.001) and MHR (HR = 1.808, 95% CI = 1.170–2.796, *p* = 0.008) and lower levels of HDL (HR = 0.558, 95% CI = 0.362–0.862, *p* = 0.009) were associated with an increased risk of mortality in patients with CJD. In addition, we found that covariates including male sex (HR = 1.770, 95% CI = 1.107–2.830, *p* = 0.017), lower Barth Index (HR = 0.642, 95% CI = 0.416–0.991, *p* = 0.035), higher NSE (HR = 1.558, 95% CI = 1.002–2.423, *p* = 0.049), hyperintensity of basal ganglia on MRI (HR = 2.085, 95% CI = 1.254–3.465, *p* = 0.005) and PSWCs on EEG (HR = 1.855, 95% CI = 1.121–3.069, *p* = 0.016) also increased the risk of mortality.

**Fig. 4:**
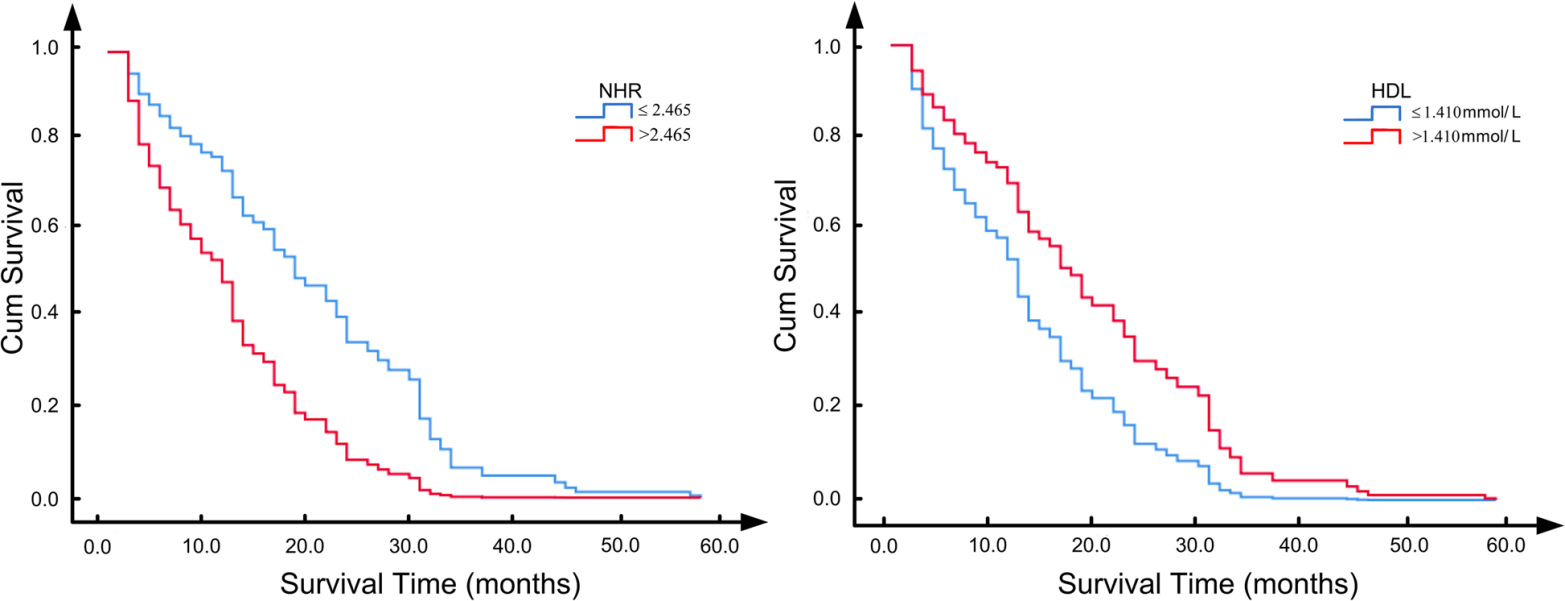
Survival curves of CJD patients with high vs low NHR and HDL levels. A median split was conducted on NHR and HDL. Cox proportional hazards regression models were adjusted for sex, Barth Index, NSE, hyperintensity of DWI/FLAIR in basal ganglia and PSWCs on EEG. NSE, neuron-specific enolase; PSWCs, periodic sharp wave complexes; HDL, high-density lipoprotein; NHR, neutrophil to high-density lipoprotein

After adjusting for sex, Barth Index, NSE, hyperintensity of DWI/FLAIR in basal ganglia and PSWCs on EEG, NHR (HR = 2.344, 95% CI = 1.277–4.303, *p* = 0.006) and HDL (HR = 0.567, 95% CI = 0.346–0.930, *p* = 0.025) were identified as independent survival factors in the multivariate Cox regression analysis. Given the collinearity of NHR and HDL, we did not involve these two factors into one multivariate model. The results are shown in Table 3 and Fig. 4.

**Table 3:** Univariate and multivariate Cox proportion hazards regression analyses for survival

	No Covariates			Covariates		
	HR	95% CI	*p* value	HR	95% CI	*p* value
**Covariates**						
sex	1.770	1.107–2.830	0.017	NA	NA	NA
age	0.903	0.589–1.382	0.637	NA	NA	NA
Barth Index	0.642	0.416–0.991	0.035	NA	NA	NA
CSF 14–3-3 protein	1.214	0.771–1.913	0.403	NA	NA	NA
NSE	1.558	1.002–2.423	0.049	NA	NA	NA
hypertension of basal ganglia on DWI	2.085	1.254–3.465	0.005	NA	NA	NA
PSWCs on EEG	1.855	1.121–3.069	0.016	NA	NA	NA
**Inflammatory biomarkers**						
NLR	1.455	0.938–2.259	0.094	0.970	0.569–1.653	0.910
HDL	0.558	0.362–0.862	0.009	0.567	0.346–0.930	0.025
NHR	3.206	1.967–5.223	< 0.001	2.344	1.277–4.303	0.006
MHR	1.808	1.170–2.796	0.008	1.300	0.795–2.125	0.295

*NSE* Neuron-specific enolase; *PSWCs* Periodic sharp wave complexes, *NLR* Neutrophil-to-lymphocyte ratio, *HDL* High-density lipoprotein, *NHR* Neutrophil to high-density lipoprotein ratio, *MHR* Monocyte to high-density lipoprotein ratio

## Discussion

To the best of our knowledge, this is the first study to explore the correlation between peripheral inflammatory biomarkers and the disease severity and prognosis in patients with CJD. We found that NLR, NHR and MHR were positively related to the disease severity, and decreased HDL and increased NHR were independent predictive biomarkers for poor prognostic outcomes in CJD patients.

Our study found that the median survival time was 12.5 months in patients with CJD. It was longer than that reported in the Caucasian population, but similar to the reported in other Chinese research [1, 27]. In another Asian country, Japan, the mean survival time was 15.7 months [28], which was longer than that in our study. We hypothesized that the difference might be related to the races and regional environments. In China, 97–100% of CJD cases were MM at codon 129 [29]. According to the PrP^Sc^ differences, MM type could be further divided into MM1 and MM2 subtypes. Patients with MM2 subtype have relatively longer survival time. A previous study found that almost all the patients with MM2 had survival time longer than one year, with the longest of 50 months [30]. A proportion of the patients in our study might be MM2 subtype. Therefore, the median survival time might be relatively longer. In addition, the longer survival time in our study might be associated with the study design. One of the factors affecting the prognosis of patients with CJD is infection. However, to minimize the influence of comorbidities on the results, we excluded patients with acute and chronic infections such as pulmonary infections and urinary tract infections. Furthermore, our study showed that two-thirds patients were male. However, the sex bias was not significant in the 178 cases we initially enrolled. Thus, we thought that the remarkable sex disproportion was generated during the screening process according to the exclusion criteria, and it did not reflect the true sex ratio of CJD.

The NLR in the peripheral blood is altered in various neurodegenerative disorders, such as ALS, MAS, AD and PD [8, 9, 31, 32]. In cerebrovascular disorders such as stroke, NLR was also found to be associated with prognosis as well as mortality outcomes [33, 34]. However, it has never been investigated in CJD previously. Usually, higher NLR values are associated with severe inflammation and worse survival outcomes. While our findings are consistent with that of other neurodegenerative diseases, the pathophysiological mechanism of NLR in CJD is unclear. Studies increasingly show that central inflammation plays an important role in the pathogenesis of CJD [4, 35–37]. Typically, prion diseases do not elicit a prominent inflammatory response in the periphery, which can be attributed to the immune tolerance on account of the similar immunogenicity of PrP^Sc^ and PrP^C^ [38]. Nonetheless, several inflammatory markers, such as CRP, IL-6, AAT, α1-acid glycoprotein and fibrinogen, are elevated in the plasma of CJD patients, suggesting that a systematic inflammatory reaction might also associated with pathogenesis of CJD [5, 6]. Neutrophils are innate immune cells that clear pathogens through multiple mechanisms, including production of reactive oxygen species (ROS), phagocytosis, degranulation and release of neutrophilic extracellular traps (NETs). Furthermore, its proinflammatory effects have been implicated in the onset and progression of neurodegeneration [39]. Native and scrapie-associated prion protein inhibit neutrophils function and down-regulate the percentage of neutrophils in peripheral blood [40, 41], thus inhibiting neutrophils aggregation and the export of superoxide radicals and beta-glucuronidase, which may reduce the tissue damage and delay disease progression. PrP^Sc^ accumulation in the CNS also release chemokines that attract peripheral activated T cells [42]. Although the underlying mechanisms need further investigation, this finding suggests that peripheral immune dysfunction might contribute to the progression of CJD.

In our study, the level of plasma HDL was associated with survival time of CJD patients. HDLs are the predominant lipoproteins in human brains that mediate cholesterol efflux and form lipid rafts that are critical for cellular signaling [11, 43]. Studies show that inadequate or dysfunctional brain HDLs contribute to neurodegeneration, as well as neurovascular instability and dysfunction [13, 14, 44]. Depletion of intracellular cholesterol or inhibition of cholesterol synthesis prevents PrP^Sc^ accumulation in neuronal cells [45, 46]. Decreased HDL levels in brain might increase PrP^Sc^ accumulation by reducing the efflux of cholesterol. Since plasma HDL cholesterol and apoA-I levels correlate with that in CSF, plasma apoA-I/HDL may reflect and even influence brain apoA-I/HDL levels [47]. Therefore, the reduction of HDL in the plasma may promote disease progression and could explain the short survival duration of the CJD patients in our study. In addition, Perrier et al. also found that elevated plasma cholesterol in mice significantly shortened their survival and markedly increased the accumulation rate of PrP^Sc^ deposits in the brain tissues, which is also consistent with our findings [48].

The HDL-related inflammatory biomarkers, NHR, was also associated with disease severity and prognosis of CJD. Previous studies have also reported that NHR correlates positively with disease severity in PD and acute ischemic stroke [16, 17]. It could be a more reliable biomarker for CJD since it combines the anti-inflammatory and antioxidant effects of HDL and the pro-inflammatory effect of neutrophils. Furthermore, HDL is known to inhibit neutrophil activation, adhesion, diffusion and migration, and a large number of activated neutrophils also could affect HDL composition and function [49, 50]. In fact, the hazard ratio of NHR in the survival analysis was higher than that of HDL, and we found that NHR but not HDL was associated with disease severity. These findings suggest that neutrophils and HDL may synergistically promote CJD pathogenesis, and NHR may reflect the inflammatory changes in CJD more accurately that neutrophil count or HDL levels alone.

In addition, NLR, NHR and MHR were also related with NSE in plasma, and MRI and EEG manifestations. Previous studies have shown that higher level of NSE in plasma, hyperintensity in basal ganglia and PSWCs on EEG mostly appear in the middle and late stages of CJD [23, 24, 51]. Thus, these results further support the potential prognostic value of peripheral inflammatory biomarkers in CJD.

Although our study demonstrated that inflammatory indicators were associated with disease severity or prognosis, the actual strength of correlations was not very high, with the correlation coefficients consistently being less than 0.5. Thus, these results should be interpreted with caution, and it could be better to combine clinical symptoms or other auxiliary examinations to evaluate. Nevertheless, these findings provided evidence that peripheral inflammation might be involved in the pathogenesis of CJD, and help us understand the association between inflammation and prion pathology.

There are several limitations in our study that ought to be considered. First, the findings of this study should be interpreted in the context of the study design and patient population. In order to exclude the influence of concomitant diseases on inflammatory indicators, we adopt strict exclusion criteria. However, pulmonary and urinary tract infections are common complications in patients with CJD, especially in the advanced disease stage. Thus, the clinical application of inflammatory biomarkers in those patients was limited. In addition, in our study, the majority of patients were sporadic CJD, and all of the them were MM genotype. Thus, the relationships between these biomarkers and prognosis in other types of CJD such as genetic CJD and patients with MV/VV subtypes need further study to verify. Second, We only estimated the baseline NLR, HDL, NHR and MHR, and the changes in these biomarkers during disease progression need to be explored further through long-term longitudinal studies. Third, most of the patients included in our study were clinically diagnosed without postmortem diagnosis. Fourth, the performance of Barth Index in evaluating disease severity is relatively poor, and the Medical Research Council (MRC) Prion Disease Rating Scale should be used in further research. Fifth, the size of the sample was relatively small and the patients were recruited from a single hospital. Thus, our results need to be validated on a larger, multi-center cohort.

## Conclusions

Inflammatory factors in the plasma are simple, inexpensive and reliable biomarkers for predicting disease severity and prognosis in patients with CJD. NHR is significantly associated with disease severity as well as survival duration. This study suggested that peripheral inflammatory responses may play a role in the pathogenesis of CJD and provide new insights into the assessment and treatment of CJD.

## Supplementary Information

**Additional file 1:** **Table 1.**The association between inflammatory biomarkers and auxiliary examinations inpatients with CJD. 

## Data Availability

The datasets generated and analyzed during the current study are not publicly available due to the identity information contained in the data but are available from the corresponding author on reasonable request by deleting this information.
