# Supplementary material for: Neutrophil to High-density Lipoprotein ratio (NHR) as a potential predictor of disease severity and survival time in Creutzfeldt-Jakob disease
Source: BMC Neurol. 2023 Jan 23;23:34. doi: 10.1186/s12883-023-03076-y (PMC9869630; doi:10.1186/s12883-023-03076-y)
Supplement: Supplementary file 1 — Additional file 1: Table 1.The association between inflammatory biomarkers and auxiliary examinations inpatients with CJD. [file 12883_2023_3076_MOESM1_ESM.docx]

**Table 1** The association between inflammatory biomarkers and auxiliary examinations in patients with CJD

| Inflammatory biomarkers | Correlation with NSE, *r* | *p* value | CSF 14-3-3 protein | | *p* value | hyperintensity of basal ganglia on MRI | | *p* value | PSWCs on EEG | | *p* value |
| --- | --- | --- | --- | --- | --- | --- | --- | --- | --- | --- | --- |
|  |  |  | (+)  n=36 | (-)  n=39 |  | (+)  n=22 | (-)  n=66 |  | (+)  n=22 | (-)  n=59 |  |
| NLR | 0.102 | 0.359 | 2.02(1.59-2.25) | 1.95(1.12-2.79) | 0.636 | 2.56(1.81-2.92) | 1.95(1.34-2.23) | 0.002 | 2.36(±0.86) | 1.91(±0.76) | 0.024 |
| HDL | -0.182 | 0.1 | 1.40(±0.34) | 1.50 (±0.40) | 0.236 | 1.18(1.05-1.55) | 1.49(1.16-1.65) | 0.73 | 1.30(±0.31) | 1.46(±0.39) | 0.083 |
| NHR | 0.341 | 0.002 | 2.33(1.93-3.12) | 2.30(1.49-2.93) | 0.266 | 3.21(2.56-3.89) | 2.24(1.59-2.93) | 0.01 | 2.97(2.48-4.02) | 2.30(1.55-3.22) | 0.005 |
| MHR | 0.284 | 0.009 | 0.24(0.18-0.33) | 0.21(0.16-0.28) | 0.144 | 0.29(0.23-0.35) | 0.23(0.17-0.29) | 0.024 | 0.29(0.22-0.35) | 0.23(0.18-0.30) | 0.055 |
